# Impact of COVID-19 on nurse outcomes in the private sector of South Africa: a cross-sectional study

**DOI:** 10.1186/s12912-024-02559-8

**Published:** 2024-12-18

**Authors:** Granny Solofelang Gohentsemang, Siedine Knobloch Coetzee, Stephani Botha, Erika Fourie

**Affiliations:** 1 NuMIQ Research Focus Area, School of Nursing Science, North-West University, Private Bag X6001, Potchefstroom, South Africa; 2 Unit for Business, Mathematics and Informatics, North-West University, Private Bag X1290, Potchefstroom, South Africa

**Keywords:** Burnout, Compassion fatigue, Compassion satisfaction, COVID-19, Nurses, Turnover intentions

## Abstract

**Background:**

This study explored and described the impact of the COVID-19 pandemic on nurse outcomes in the private sector of South Africa. National research shows that nurses had poor nurse outcomes prior to the pandemic, amidst these issues the COVID-19 pandemic occurred, with nurses having to play a key role in the public health response. International studies have shown that although nurses were willing to serve in this manner, they experienced moderate to high burnout, anxiety, depression, fear and exhaustion. However, this topic has received comparatively little attention in African countries.

**Methods:**

A cross-sectional survey design was conducted. Multistage sampling was applied in selection of provinces, hospital groups, hospitals, units, and nursing personnel. Valid and reliable tools were used to measure nurse outcomes. Data was collected from April 2021 until January 2022.

**Results:**

Nurses described having high levels of compassion satisfaction, moderate levels of compassion fatigue, and high levels of burnout. Nurses appeared satisfied with their job and career. Almost a quarter of nurses reported the intention to leave their job, and of those about a fifth indicated that they intended to leave the profession. The nurses who routinely cared for COVID-19 patients had a small statistically significant increase in compassion fatigue, compassion satisfaction, emotional exhaustion and, job turnover intention, and a small statistically significant decrease in job satisfaction. Increased exposure to death and dying showed small correlations with emotional exhaustion and career turnover intentions.

**Conclusion:**

The results of this study show that nurses were impacted by COVID-19 and death and dying during the pandemic, and that follow-up studies are needed post-pandemic.

**Implications of study:**

It is important that burnout and compassion fatigue be addressed on an organizational level, as nurse outcomes were already negative prior to the pandemic, and all global evidence points to the worsening of these outcomes post-pandemic. There were many psychological support interventions with proven effectiveness that should be explored and applied for the South African context.

## Key points

• Nurses in developed countries report poor nurses’ health and career outcomes during the COVID-19 pandemic, this study confirms similar results in South Africa.

• Regular exposure to COVID-19 patients had small associations with compassion satisfaction, compassion fatigue, emotional exhaustion, job turnover intention, job satisfaction and career turnover intention.

• Regular exposure to death and dying had small associations with emotional exhaustion and career turnover intentions.

## Background

National research that was conducted between 2009 and 2012 in South Africa (SA) has shown that nurses are not satisfied with their jobs, especially with regard to their salaries, workload and the resources available to them [1–3]; that they rated the practice environment as poor or fair, they were burned out [2], and intended to leave the profession [2–4]. In this national study, nurse outcomes were worse in the public sector than in the private sector [1, 2]. Cummings et al., 2010 defines nurse outcomes as consisting of four areas namely satisfaction with work (e.g. job satisfaction, career satisfaction), relationship with work (e.g. intention to leave job, intention to leave profession), health and wellbeing (e.g. burnout, physical and mental health) and productivity and effectiveness (e.g. work performance, job involvement). The South African Presidential Health Summit aimed to address several of these problems, but amidst these issues the COVID-19 pandemic occurred, with nurses having to play a key role in the public health response [5].

Nurses were on the frontline of the COVID-19 pandemic, delivering direct patient care, providing emotional support to patients in the absence of social support networks, witnessing an increased incidence of illness and death, and risking personal exposure to an infectious disease [6]. International studies have shown that although nurses were willing to serve in this manner, they experienced moderate to high burnout, anxiety, depression, fear [6] and exhaustion [7]. However, this topic has received comparatively little attention in African countries.

COVID-19 was first identified in an outbreak in Wuhan city in the Hubei Province of China [7]. The World Health Organization (WHO) declared COVID-19 a public health emergency of international concern at the end of January 2020, and it was declared a global pandemic on 11 March 2020 [8]. According to the South African Government News Agency, the first incidence was confirmed on 5 March 2020 and the country proceeded to declare a state of disaster on 15 March 2020 [9]. Approximately 2 weeks thereafter a nationwide stay-at-home total lockdown was declared, beginning at midnight on 26 March 2020 [8]. This was effective for 21 days, and later extended [8]. Declaration of the state-wide lockdown took place as SA’s confirmed case count had grown from 61 to 402 cases in only eight days [9]. Traveling was restricted, gatherings were prohibited, and only essential services (inclusive of food production, distribution and sales of food items, pharmacies and medical facilities) were given permission to remain open [10]. Furthermore, COVID-19 lockdowns did not apply to essential workers such as police, traffic officers, and healthcare workers (HCWs) [9].

The main issues facing nurses globally in response to the COVID-19 pandemic were personal protective equipment (PPE), job content, work organization, and social context [11]. The social context included outbursts of uncertainty, acute panic, fear, depression, obsessive behaviors, social unrest, stigmatization, anxiety, increased incidence of gender-based violence, and discrimination in the distribution of emergency food aid [12]. The nature of COVID-19 had severe and negative effects on healthcare practitioners’ health, survival, emotional state, and coping strategies [13].

There is an abundance of research on the health and wellbeing of nurses in South Africa prior to the pandemic [2–22]. However, since the beginning of COVID-19 research studies on this topic have been carried out only in single provinces or hospitals [24–26], showing high levels of depression, anxiety and post-traumatic stress. The Human Sciences Research Council (HSRC) in South Africa emphasized that more nurses than other HCWs reported serious concerns about self-perceived risk of contracting COVID-19, concern for personal and family well-being, concern for passing infection to family members and general health and well-being. Such concerns affect nurses’ capabilities, performance and morale. Emotional distress was significantly higher among public sector HCWs compared to their private sector counterparts [27]. This is probably because about 84% of our population is reliant on the public health sector, while the HCW have the least concentration of HCWs.

Beyond the initial rapid reaction to the effects of COVID-19, there are longer-term difficulties to consider: systems must now assist immunization campaigns, keep up with additional and upcoming pandemic waves, and consider how to handle increases in demand brought on by infected patients who now have long-term chronic diseases [11, 28]. Nurses and their coworkers had to determine how much care they could give to others in times of pandemics, while still taking care of themselves [29]. To address the backlog of non-COVID-19 treatment, it is also necessary to redirect resources to other areas of the healthcare system [11]. In most nations, the demand-supply imbalance for nurses has grown because of the interaction of several demand-side factors [11]. The viability of the nursing workforce depends on resolving these critical concerns [11]. The epidemic has compelled swift policy adjustments to boost nurse supply at system level in all nations; however, focusing just on the system level and ignoring the impact on nurses will result in worsening nurse retention [11]. It was therefore necessary to explore nurse outcomes in South Africa during the pandemic, to respond to these as the long-term repercussions of COVID-19 become apparent and in preparation for future healthcare crises. The aim of the study was to explore and describe the impact of COVID-19 on nursesoutcomes in the private sector of SA.

## Methods

### Design and respondents

This quantitative study applied a cross-sectional design using a self-report online survey [30]. Multistage sampling was applied in the selection of the provinces, hospital groups, hospitals, units, and nursing personnel in this study. Data were collected in all nine provinces of SA, namely North West, Northern Cape, Western Cape, Eastern Cape, Gauteng, Limpopo, KwaZulu-Natal, Mpumalanga, and Free State. The South African health sector consists of a public and private healthcare sector. The public sector has five different tiers depending on care provided, namely central, tertiary, regional, district and specialist hospitals [31], and in the private sector there are five main private hospital groups [32]. Only the four largest private hospital groups wer taken into consideration in this study, as the largest three dominate private sector beds, and theatre facilities, but the fourth is still a notable player.

To limit the risk of COVID-19 infection and research participation burden on essential healthcare practitioners, online data collection was advised. The private sector was consulted, and each hospital group (*n* = 4) confirmed that it had the necessary resources and communication platforms to assist with an online self-report survey. All hospitals (*n* = 116), except specialist hospitals, were included in the study. All in-patient units, including theatres and the emergency departments of the hospitals, were included. All nursing personnel (registered nurse and/or midwife (RN/M) (*n* = 1493), community service nurses (CSN) (*n* = 8), enrolled nurses and midwives (EN/M) (*n* = 336), and enrolled nursing auxiliaries (ENAs) (*n* = 413), including fixed- term contract and agency staff, were included. Agency staff were included as many retired nurses, as well as nurses from external agencies were recruited to work on a more permanent basis (exceeding 3 months) due to the COVID-19 pandemic. Nursing staff that indicated having less than 3 months experience in the unit, were removed from data analysis. The sample size was based on planned statistical analyses, as recommended by Maree et al. [33] because a true random sample was not possible.

In order to be eligible to participate in the study, respondents had to have worked in the chosen unit/hospital for at least three months. Student nurses were also excluded, as they often rotate among units and hospitals. The online surveys took approximately 30–45 min to complete. The researchers were not physically available during completion of the surveys, but contact information was provided in case respondents needed to ask any questions or gain clarification.

## Data collection

This is a sub-study that falls under a larger research study with the title ‘Nurse outcomes, patient safety and quality of care in the public and private hospitals of South Africa’ (NWU-00033-19-A1). The sub-study is titled ‘Impact of COVID-19 on nurse outcomes in the private sector of South Africa’ (NWU-00033-19-A1-03). The study was approved by the North-West University Health Research Ethics Committee (NWUHREC), and ethics committees of the four private hospital groups.

Data were collected during the period 2021–2022. after the second wave of the COVID-19 pandemic, from 18 April 2021, and continued to 31 January 2022. Goodwill consent was granted by each participating hospital group. The principal investigator (PI) contacted the hospital group nurse executive or delegated mediator to discuss the proposed data collection plan. The role of the mediator in this study was to facilitate communication between the researcher, unit managers and respondents in explaining the research study. Furthermore, the mediator’s ideas and remarks were welcomed in terms of the practicality and feasibility of the proposed data collection plan, to suit each individual private hospital group. The mediator was asked to help identify an independent person in each private hospital group, whose role in this study was to assist with distribution of the survey link to potential respondents.

The Uniform Resource Locator (URL) for the survey was sent to the independent person by the PI to be distributed internally by the respective private hospital group. The study was conducted anonymously, and therefore the researcher had no access to personal information of the potential respondents. By clicking on the link provided, respondents were directed to the SurveyMonkey server as the administrative tool. Respondents were required to follow the URL/link to access a page with the informed consent form, then to click on either ‘Accept’ to continue to the survey or ‘Decline’ to exit the site. The informed consent form preceded the survey, providing all project details as well as the researcher’s contact details in case the respondents have any questions. All the respondents had to give informed consent in order to continue with the survey. The survey responses were independently downloaded by the PI on Excel for further analysis. The survey data could not be accessed by the respective hospital groups. Only the PI had access to the data and coded it thereafter. Surveys were coded for data analysis at hospital and unit level, in order to identify which unit, the data was from, as the practice environments of each unit are unique (e.g. Intensive Care Unit (ICU), maternity ward, etc.). At the end of the survey, there was a lucky draw for those who wanted to enter. Only a cellular phone number was required to enter for the draw; however, it was not compulsory. The cellular number were not for research purposes, but solely to contact and inform the winners.

This study was part of a larger study and used a self-report, online survey comprising of standardized instruments used to measure the variables identified as indicated in Table 1. Most of the instruments were available in the public domain, but where relevant, permission was obtained from the developers of the instrument. No changes were made to the instruments.

**Table 1:** Standardized instruments used to measure the variables in this study

Instruments	Items	Measures	Validity and reliability
**Compassion fatigue** The compassion practice instrument (CPI) measures compassion fatigue and compassion satisfaction [35] Sample item ‘I struggle to open myself up to patients suffering and sorrow.’	Compassion satisfaction: 5 Compassion fatigue: 15 • Disengaged: 6 • Impotent: 4 • Unfulfilled: 5 Likert Scale: Range (1) Never, to (6) Always	CPI consists of 20 items and employs a 6- point scale ranging from ‘Never’ (1) to ‘Always’ (6). A mean score of 1–2 is indicative of low levels of compassion fatigue, while 2.1–4 indicates medium levels of compassion fatigue, and 4.1–6 indicates high levels of compassion fatigue. Regarding compassion satisfaction, a mean score of 1–2 is indicative of low levels of compassion satisfaction, while 2.1–4 indicates moderate levels of compassion satisfaction, and 5–6 high levels of compassion satisfaction	The CPI is a valid and reliable measure for compassion fatigue and compassion satisfaction in the South African context [36]. CFA supported a 4-factor structure. This instrument has demonstrated good construct validity [35]. **Cronbach’s alpha reliability** Compassion satisfaction: α = 0.84 Compassion fatigue: α = 0.77 Disengaged: α = 0.75 Impotent: α = 0.84 Unfulfilled: α = 0.64
**Burnout** The Maslach Burnout Inventory (MBI) measures emotional exhaustion, depersonalization and personal accomplishment [37] Sample item ‘I feel emotionally drained from my work.’	Emotional exhaustion: 9 Likert Scale: Range (1) Never, to (7) Every day Total scores are used to determine high (27 or above), medium (17–26) and low (0–16) levels of emotional exhaustion	Only the emotional exhaustion portion of the MBI was used to test for burnout. Burnout was defined as a high level of emotional exhaustion where the total score is greater than 27.	This instrument has demonstrated good validity and reliability (3). In this study, the subscale had a Cronbach alpha of 0.89
**Job satisfaction** **Registered Nurse Forecast** (**RN4CAST**) Measures overall job and career satisfaction, and 10 items regarding specific aspects of job satisfaction. Sample item ‘How satisfied are you with your job?’	Job satisfaction: 11 questions *10 items* 1) Work schedule 2) Opportunities for advancement 3) Independence at work 4) Professional status 5) Salary/wages 6) Educational opportunities 7) Appreciation, recognition and rewards 8) Immediate supervisor/ manager 9) Co-workers 10) Work-life balance Likert Scale: (1) Very dissatisfied, to (4) Very satisfied	The mean and standard deviation is reported.	Used as single items in RN4CAST studies in Europe and the United States of America (USA) [38] and South Africa (2)
**Career satisfaction** **RN4CAST** Measures overall job and career satisfaction	Career satisfaction: 1 • How satisfied are you with your nursing career? Likert Scale: Range(1) Very dissatisfied, to (4) Very satisfied	The mean and standard deviation are reported	Used as single items in RN4CAST studies in Europe and the USA [38], and South Africa (2)
**Absenteeism**	In the past year, how many times have you missed work? In days:	The mean and standard deviation are reported	Used as single items in RN4CAST studies in Europe and the USA [38], and South Africa (2)
**Job and career turnover intent** **RN4CAST** Do you plan on leaving your job in the next year? If yes, what type of work would you seek?	Overall job and career turnover intent: 2 1 = Yes, or 2 = No 1 = Nursing in another hospital; 2 = Nursing, but not in a hospital; 3 = Non-nursing career	The percentage of respondents that indicated job turnover intent (selected ‘Yes’) and the percentage that indicated career turnover intent (selected ‘Non-nursing career’)	Used as single items in RN4CAST studies in Europe and the USA [38], and South Africa (2)
**COVID-19**	How often in your current practice setting do you have to directly care for COVID-19 patients? 1 (Never), to 4 (Routinely)	Hierarchical linear modelling (multi-level modelling) using Spearman’s rank-order correlations were applied to determine the correlations per variable	Item developed from the larger study
**Death and dying**	How often in your current practice setting do you have to deal with death and dying? 1 (Never), to 4 (Routinely)	Hierarchical linear modelling (multi-level modelling) using Spearman’s rank-order correlations were applied to determine the correlations per variable	Item developed from the larger study

### Data analysis

The software program SPSS version 27 [34] was used to analyze the data. The demographic information of the individuals was presented using descriptive statistics. To make sure the measures were valid and reliable in the South African context, confirmatory factor analyses were carried out in the larger study, and these results are reported in this study. The mean replacement method was used to account for missing data.Hierarchical linear modelling (multi-level modelling) using Spearman’s rank-order correlations was applied to determine the correlations and associations between COVID-19, death and dying, and nurse outcomes. This method recognizes the dependency of data from units of a specific hospital by clustering hospital units within hospitals when determining relationships between variables. The magnitude of correlations is regarded as the effect size, where 0.1 is considered as small, 0.3 as medium, and 0.5 as large.

## Results

Of the 2260 respondents, most were female (92.5%; *n* = 2088) and employed full-time (93.6%; *n* = 2111). The majority were registered nurses (66.4%; *n* = 1439) and 21.4% (*n* = 479) of these had a bachelor’s degree in nursing. About a quarter of the nurses (25.2%; *n* = 569) indicated having a nursing specialty, with speciality training at a postgraduate diploma (56.7%; *n* = 321). Most respondents worked in a critical care unit (21.4%; *n* = 481), surgical unit (20.3%; *n* = 456) or medical unit (17.5%; *n* = 393) (see Table 2).

**Table 2:** Demographic characteristics of respondents (*n* = 2260)

Demographics		Frequency (*n*)	Percentage (%)
Gender	Female	2088	92.5
	Male	169	7.5
Employment status	Full-time	2111	93.6
	Part-time	27	1.2
	Contract	33	1.5
	Agency	84	3.7
Nursing category	RN	1493	66.4
	Community service	8	0.4
	EN	336	14.9
	ENA	413	18.4
Bachelor’s degree	Yes	479	21.4
	No	1022	78.6
Specialty training	Yes	569	25.2
	No	1690	74.8
Level of specialty training	Certificate	164	29.0
	Postgrad diploma	321	56.7
	Honours	46	8.1
	Master’s	31	5.5
	PhD	4	0.7
Specialty area of current unit	Medical	393	17.5
	Surgical	456	20.3
	Trauma	156	6.9
	Maternity	160	7.1
	Critical care	481	21.4
	Theatre	197	8.8
	Pediatrics	129	5.7
	Other	22	1.0
	Neonatal ICU	69	3.1
	Management	101	4.5
	Education	45	2.0
	Psychiatry	20	0.9
	Oncology	19	0.8
	COVID	3	0.1
Provinces	Eastern Cape	53	2.4
	Free State	78	3.5
	Gauteng	1002	44.8
	KwaZulu-Natal	452	20.2
	Limpopo	84	3.8
	Mpumalanga	40	1.8
	North West	8	0.4
	Northern Cape	38	1.7
	Western Cape	484	21.6
Death and dying	Never	65	3.2
	Rarely	542	26.9
	Occasionally	978	48.5
	Routinely	432	21.4
COVID	Never	122	6.1
	Rarely	299	14.8
	Occasionally	755	37.5
	Routinely	840	41.7
Descriptives	N	Mean	Std deviation (SD)
Age	2244 (20–71 years)	42.26	10.23
Yrs worked as nurse	2247	16.44	11.53
Yrs worked in hospital	2260	8.22	7.10

The majority were from Gauteng (44.8%; *n* = 1002), Western Cape (21.6%; *n* = 484), and KwaZulu-Natal (20.2%; *n* = 452). The majority of respondents reported occasionally and routinely caring for COVID-19 patients (79.2%; *n* = 1595), and having occasional and routine exposure to death and dying (69.9%; *n* = 1410). Their ages ranged from 20 to 71 years, with an average age of 42.26 years (SD = 10.23). Respondents had worked on average 16.44 years (SD = 11.53) as nurses and 8.22 years (SD = 710) in the specific hospital where they were working at the time of the study (see Table 2).

Nurses reported high compassion satisfaction (M = 5.30, SD = 0.82), moderate compassion fatigue (M = 2.19, SD = 0.79), and high levels of burnout (emotional exhaustion) (M = 29.59, SD = 14.34). In terms of compassion fatigue, nurses felt moderately disengaged (M = 2.33, SD = 0.92), impotent (M = 2.19, SD = 1.01) and unfulfilled (M = 2.04, SD = 0.88).

Generally, nurses reported being satisfied with their nursing career (M = 3.49, SD = 0.80) and to some degree satisfied with their job (M = 3.03, SD = 0.85). In terms of job satisfaction nurses were satisfied with certain aspects of their job such as professional status (M = 3.25, SD = 0.82), independence at work (M = 3.21, SD = 0.79), and work schedule (M = 3.17, SD = 0.89). However, respondents were dissatisfied with salary/wages (M = 2.23, SD = 0.99), appreciation, recognition and rewards (M = 2.42, SD = 1.01) and educational opportunities (M = 2.75, SD = 0.98). Absenteeism was reported to be 5.93 days (SD = 11.25) in a year, mostly due to sick leave and family responsibility leave. Most nurses (76.2%; *n* = 1672) had no intentions of leaving their job, although of those intending to leave their job 52.3% (*n* = 281) intended to seek nursing work at another hospital and 26.3% (*n* = 521) intended seeking another nursing job outside the hospital settings Almost a quarter of this number (21.4%; *n* = 115) intended leaving the nursing profession altogether (See Table 3).

**Table 3:** Descriptive variables (*n* = 2260)

Variables	Score Range	Mean	SD	Reliability	Percentage (%) with high levels
**Compassion practice**					
Compassion satisfaction	1–6	5.30	0.82	0.84 α	78.5% (1887)
Compassion fatigue	1–6	2.19	0.79	0.77 α	3.45% (67)
Disengaged	1–6	2.33	0.92	0.75 α	
Impotent	1–6	2.19	1.01	0.84 α	
Unfulfilled	1–6	2.04	0.88	0.64 α	
**Burnout**					
Emotional exhaustion	1–7	29.59	14.34	0.89 α	33.57% (608)
**Job satisfaction**					Percentage (%) very dissatisfied
How satisfied are you with your job?	1–4	3.03	0.85	Single item	7.4% (158)
Work schedule	1–4	3.17	0.87	Single item	6.6% (141)
Opportunity for advancement	1–4	2.81	0.96	Single item	13.4% (287)
Independence at work	1–4	3.21	0.79	Single item	4.5% (96)
Professional status	1–4	3.25	0.82	Single item	5.0% (106)
Salary/wages	1–4	2.23	0.99	Single item	30.6% (657)
Educational opportunities	1–4	2.75	0.98	Single item	14.9% (320)
Appreciation, recognition and rewards	1–4	2.42	1.01	Single item	24.2% (519)
Immediate supervisor/ manager	1–4	3.06	0.93	Single item	8.8% (188)
Co-workers	1–4	3.1	0.75	Single item	3.9% (83)
Work-life balance	1–4	2.87	0.88	Single item	9.1% (196)
**Career satisfaction**					
How satisfied are you with your nursing career?	1–4	3.49	0.80	Single item	4.4% (95)
**Absenteeism**					
Absenteeism	0-150	5.93	11.25	Single item	
**Job turnover intentions**					
Do you plan on leaving your job in the next year?	Yes		No		
	521 (23.8%)		1672 (76.2%)		
**Career turnover intentions**					
If yes, what type of work would you seek?	Nursing in another hospital		Nursing, but not in a hospital	Non-nursing career	
	281 (52.3%)		141 (26.3%)	115 (21.4%)	

There were small, statistically significant positive relationships between COVID-19 and nurse outcomes (See Table 4). The following small positive correlations can be highlighted: overall compassion fatigue (*r* = 0.061**; p = < 0.0001), and its two dimensions (disengaged [*r* = 0.071**; p = < 0.0001] and unfulfilled [*r* = 0.059**; p = < 0.0001]), compassion satisfaction (*r* = 0.054**; p = < 0.0001), job turnover intention (*r* = 0.063**; p = < 0.0001 and emotional exhaustion (*r* = 0.100**; p = < 0.0001). The following small negative correlations can be highlighted: overall job satisfaction (*r*= -0.061**; p = < 0.0001) and immediate supervisor/manager (*r*= -0.084**; p = < 0.0001).

**Table 4:** Correlations between variables

Variables		COVID 19 (Never = 1; Rarely = 2; Occasionally = 3; Routinely = 4)	Death and dying (Never = 1; Rarely = 2; Occasionally = 3; Routinely = 4)
Compassion fatigue	Correlation coefficient	0.061	0.044
	Sig. (2-tailed)	< 0.0001	< 0.0001
	N	2010	2011
Compassion satisfaction	Correlation coefficient	0.054	-0.037
	Sig. (2-tailed)	< 0.0001	< 0.0001
	N	2011	2012
Disengaged	Correlation coefficient	0.071	0.005
	Sig. (2-tailed)	< 0.0001	0.2736
	N	2010	2011
Impotent	Correlation coefficient	0.027	0.054
	Sig. (2-tailed)	< 0.0001	< 0.0001
	N	2009	2010
Unfulfilled	Correlation coefficient	0.059	0.052
	Sig. (2-tailed)	< 0.0001	< 0.0001
	N	2009	2010
MBI Emotional exhaustion	Correlation coefficient	0.100	0.087
	Sig. (2-tailed)	< 0.0001	< 0.0001
	N	1793	1793
Job satisfaction			
How satisfied are you with your job?	Correlation coefficient	-0.061	-0.012
	Sig. (2-tailed)	< 0.0001	0.0006
	N	2005	2005
Work schedule	Correlation coefficient	-0.021	0.001
	Sig. (2-tailed)	< 0.0001	0.8433
	N	2003	2004
Opportunity for advancement	Correlation coefficient	-0.038	0.034
	Sig. (2-tailed)	< 0.0001	< 0.0001
	N	1997	1997
Independence at work	Correlation coefficient	-0.008	-0.004
	Sig. (2-tailed)	0.0931	0.3715
	N	2000	2000
Professional status	Correlation coefficient	-0.030	0.004
	Sig. (2-tailed)	< 0.0001	0.1983
	N	1986	1985
Salary/wages	Correlation coefficient	-0.040	0.031
	Sig. (2-tailed)	< 0.0001	< 0.0001
	N	2008	2009
Educational opportunities	Correlation coefficient	-0.004	0.070
	Sig. (2-tailed)	0.4001	< 0.0001
	N	2002	2003
Appreciation, recognition, and rewards	Correlation coefficient	-0.040	0.014
	Sig. (2-tailed)	< 0.0001	0.0001
	N	2005	2005
Immediate supervisor/manager	Correlation Coefficient	-0.084	-0.038
	Sig. (2-tailed)	< 0.0001	< 0.0001
	N	2001	2001
Co-workers	Correlation coefficient	-0.005	0.005
	Sig. (2-tailed)	0.2057	0.1382
	N	2010	2010
Work-life balance	Correlation coefficient	-0.030	-0.002
	Sig. (2-tailed)	< 0.0001	0.5784
	N	2007	2007
Career satisfaction	Correlation coefficient	-0.023	0.033
	Sig. (2-tailed)	< 0.0001	< 0.0001
	N	2001	2002
Absenteeism	Correlation coefficient	0.017	0.023
	Sig. (2-tailed)	< 0.0001	< 0.0001
	N	1577	1577
Job turnover intentions	Correlation coefficient	0.063	-0.002
	Sig. (2-tailed)	< 0.0001	0.5952
	N	2008	2008
Career turnover intentions	Correlation coefficient	-0.047	-0.054
	Sig. (2-tailed)	< 0.0001	< 0.0001
	N	498	500

Correlations of 0.1 is small; 0.3 medium; and 0.5 large

There were only small, statistically significant relationships between exposure to death and dying and nurse outcomes. The following small positive correlations can be highlighted: emotional exhaustion (*r* = 0.087**; p = < 0.0001), impotent (*r* = 0.054**; p = < 0.0001), unfulfilled (*r* = 0.052**; p = < 0.0001), and educational opportunities (*r* = 0.070**; p = < 0.0001). Only career turnover intention (*r*= -0.054**; p = < 0.0001) can be highlighted with small negative correlations.

There was a small statistically significant relationship between specialty training and exposure to the independent variables of exposure to death and dying (d = 0.196**; *p* = 0.000), with those having specialty training having more exposure. There was a medium statistically significant positive relationship between the specialty of the unit and exposure to the independent variables of exposure to death and dying (d = 0.352**; *p* = 0.000) and COVID-19 (d = 0.331**; *p* = 0.000), with those in the emergency unit having the most exposure. There was a statistically significant medium negative correlation between exposure to death and dying with age (*r*=-0.289**; *p* = 0.000); and years worked in the hospital (*r*=-0.398**; *p* = 0.000), and statistically significant positive correlations with years worked as a nurse (*r* = 0.410**; *p* = 0.000) (See Table 4).

## Discussion

To our knowledge, this is the first study focusing on the impact of COVID-19 on nurse outcomes in the private sector of SA. According to the results of this study, respondents described having high levels of compassion satisfaction and moderate levels of compassion fatigue [39]. Respondents had high levels of burnout; this is in line with a previous national study [2] as well as studies conducted in the pandemic although focused on a single province in South Africa [24], but also global trends [11]. Respondents were satisfied with their job and career. The aspects of their job that they were most satisfied with were professional status, independence at work and work schedule; however, they were most dissatisfied with salary/wages, appreciation, recognition and rewards, and educational opportunities. Sick leave and family responsibility leave were reported to be the greatest causes of respondents’ absenteeism. Only a few respondents considered leaving their job and their nursing career as a profession; most would rather change from one hospital to the other or seek a nursing job outside of the hospital setting. Those with specialty training and working in the emergency units had more exposure to death and dying, and nursing of COVID-19 patients. Younger respondents with less experience expressed having higher exposure to death and dying.

The results show very little association between increased exposure to COVID-19 and nurse outcomes. According to a study conducted in China, nurses caring for COVID-19 patients experienced three stages of reactions: ambivalence toward their role (e.g. patient care vs. infection concern), emotional exhaustion (e.g. anxiety, depression, fear), and psychological adaptation [40]. Compared to before the COVID-19 outbreak, scores for compassion satisfaction and compassion fatigue in an Iranian study did not change significantly [41]. Nurses in Iran and Italy described having a moderate level of compassion satisfaction and compassion fatigue before and during the COVID-19 pandemic [41, 42]. A systematic review carried out internationally further reported a moderate level of compassion satisfaction and compassion fatigue during the COVID-19 pandemic [43], and this is in line with the results of a province-based study in South Africa by Ndlovu et al. which indicated low to moderate compassion satisfaction and moderate compassion fatigue [23].

However, in this study respondents described having higher levels of compassion satisfaction, and moderate levels of compassion fatigue. In terms of compassion fatigue, feeling disengaged and unfulfilled were related to COVID-19, while impotence and unfulfillment were related to death and dying. Respondents might have felt disengaged from patients due to the nature of COVID-19 illness and its restrictions, such donning and doffing personal protective equipment and practicing physical distancing to limit the spread of illness, and as a result felt unfulfilled. The respondents may have felt impotent after seeing the deaths of employees, family members, and patients; they believed that no matter what they tried, people died, which left them feeling unfulfilled [44, 45].

Compassion satisfaction is linked with well-being, fulfillment, reward, accomplishment, joy, enrichment, invigoration, inspiration, revitalization, gratitude and hope [46]. In this study respondents had high levels of compassion satisfaction, higher than levels reported in systematic and meta-analysis studies conducted prior to the pandemic [47–49]. Compassion satisfaction is determined by the work environment, patients’ needs and personal characteristics [47], and is most often predicted by meaningful recognition, job satisfaction, age between 50 and 65 years, fewer years of experience [47], the nursing sub-specialty of intensive care nursing [48]. Therefore the most probable reason for the high incidence of compassion satisfaction in this study could be due to the fact that respondents felt their job was valued and acknowledged during the pandemic, and they were satisfied with their job [47–49].

Respondents described having high levels of burnout. These findings related to international trends of burnout in nursing covering the pre-pandemic period and concluded that the patterns identified by these studies consistently show that adverse job characteristics, such as high workload, low staffing levels, long shifts, and low control, are associated with burnout in nursing [11]. A systematic review among sub-Saharan African nurses in the pre-pandemic period had also reported high level of burnout [50]. Up to two-thirds of nurses reported emotional exhaustion because of nursing workforce shortages [50]. Regardless of feeling valued and acknowledged during this crisis, COVID-19 put a lot of strain on nurses, over and above what they already carried [51, 52]. Respondents in this study reported a high level of emotional exhaustion, that increased with greater exposure to COVID-19 and rise in mortality rates, similar to the findings of an international studies [53–55], South African literature [23, 24] and a systematic reviews conducted during the pandemic which showed an increase in burnout, specifically the dimensions of emotional exhaustion and depersonalization, an increase in compassion fatigue, and a decrease in personal accomplishment with no real change or a decrease in compassion satisfaction [56–58]. These studies specifically found these variables to be related to intrapersonal factors and organizational factors (i.e., nurses involved in developing policies to prepare for COVID-19 patients, organizational support, and personal protective equipment [PPE] provisions) [57].

Literature has reinforced the need to look at nurse burnout and compassion fatigue as a symptom that requires organizational responses, since both burnout and compassion fatigue result in emotional distress, pain, and suffering, increased rates of absenteeism, reduced patient satisfaction, deteriorating safety and quality of care, decreasing organizational commitment and productivity, and high attrition rates and intention to leave [59, 60]. More pressing is the fact that on a global scale, Sub-Saharan Africa reports the highest leves of burnout.

Overall, in this study respondents were satisfied with their job and their career. Respondents were satisfied with certain aspects of their job, whereas a third reported feeling dissatisfied, particularly with salary, recognition and appreciation, and educational opportunities; this supports the results of a study carried out in South Africa prior to the pandemic [2–4] and internationally [61]. However, COVID-19 negatively correlated with job satisfaction, and also with the immediate supervision of managers. Those with more contact with COVID-19 patients described having more job satisfaction and were more satisfied with their supervisors. This indicates that they may have felt that their jobs made a difference, and that supervisor input was appreciated in wards with more exposure to COVID-19 with regard to overseeing medical facilities, managing human and material resources, improving productivity, efficiency, sustainability, and minimizing risk [62, 63].

Janse van Rensburg et al. [13] emphasized the need for psychological support among nurses to enhance their mental wellbeing during the COVID-19 pandemic. In fact, a recent systematic review makes a plea, that although declining nurse outcomes were seen in previous outbreaks, including SARS and A/H1N1, there were no pandemic specific interventions for COVID-19, however there is now mounting evidence of various psychological support interventions that were provided online, of which the results demonstrate the effectiveness of psychological support interventions in lowering stress and burnout while fostering self-efficacy and wellbeing among both informal carers and healthcare professionals [64]. Buchan et al. [11] further highlight that these interventions should not only have been in place during the pandemic, but also beyond for future pandemics. In addition that state that employers and organisations must accept responsibility, create supportive circumstances and develop policy interventions that concentrate on better work environments, maintaining enough staffing levels, and offering appealing working conditions, compensation, and career prospects.

Those with more contact with COVID-19 patients were more likely to intend to leave their job. The findings of this study are similar to an international integrative analysis of pre- and post-COVID-19 research on nurses’ turnover intentions, which concluded that the pandemic increased the intention rates for nurses to leave their job [11]. In this study, respondents considered rather changing hospitals or looking for jobs outside the hospital setting, but remaining in their nursing career. Nurses might have considered leaving their jobs due to fear of exposing their loved ones to the risks of contracting COVID-19 [65, 66]. Moreover, the higher reported rate of nurses’ deaths resulting from COVID-19 illness might have also impacted the intention to leave their job [67]. In other studies, a shortage of resources was considered to be the risk factor associated with nurses’ turnover intention [11, 12, 54, 68, 69], but this topic needs to be explored further. Job satisfaction is the most influential factor in increasing nurse retention, consistent with international trends [70, 71].

During the COVID-19 outbreak all patients in all units were treated as COVID-positive patients or patients under investigation [45]. Specialty training, specialty unit, and experience were positively associated with death and dying during COVID-19. Nurses working in emergency units were more affected by COVID-19 than counterparts in other units, consistent with the findings of other studies [68, 72, 73]. The results of this study, along with those of other studies, suggest that nurses who provided direct care to patients with COVID-19 illness were affected the most. These results are in line with studies conducted in South Africa [23], Ethiopia [49], and Nigeria [74]. Young nurses with less experience have not experienced and witnessed such an increased mortality rate, so death and dying of COVID-19 patients affected them more than it did the older and more experienced nurses [57] these results are in line with of the study.

The reason for small correlations could be due to the use of avoidance as a coping strategy [75] during a crisis (the COVID-19 pandemic). During avoidance coping strategies, respondents are in survival mode and coping, and not dealing effectively with their emotions [75]. As a result, they may experience delayed post-traumatic stress disorder (PTSD). This is in line with literature which associated PTSD with avoidance as a coping strategy [53, 76].

The limitations of this study included the inability to determine causality between COVID-19 and nurses’ outcomes due to the cross-sectional nature of the study. With online self-report there is no guarantee that the survey was answered by relevant nursing staff; although, the respondents had to check the box stating that they meet the inclusion criteria – that they have worked for three months or more in that hospital/unit, and they are not a student nurse.

Other literature reported that the COVID-19 pandemic was associated with staffing, resources, equipment, organizational change and psychological factors, and it is clear that those had an effect on nurses’ outcomes, but these were not tested as this was beyond the scope of this study. These factors need to be explored further. The study was conducted during the second surge of the COVID-19 pandemic, which might have influenced the nurses’ true reflection of emotions; therefore, a study on the long-term effect of COVID-19 is recommended.

## Conclusion

The results of this study show that nurses were impacted by COVID-19 and death and dying during the pandemic, and that follow-up studies are needed post-pandemic. Increased exposure to COVID-19 and death and dying, had small associations with nurse outcomes. COVID-19 had a small association with compassion satisfaction, compassion fatigue, job turnover intentions, and job satisfaction, and burnout (emotional exhaustion). Death and dying had small associations with emotional exhaustion, compassion fatigue, educational opportunities and career turnover intention. The findings of this study show that nurses experienced high levels of burnout, moderate levels of compassion fatigue and some turnover intent, especially with regard to leaving the current job. Nurses in this study did, however, experience high levels of compassion satisfaction and job satisfaction. It is important that burnout and compassion fatigue be addressed on an organizational level, as global trends, systematic reviews and meta-analysis report that nurse outcomes worsened during the pandemic and post-pandemic, with urgent intervention needed.

### Relevance of clinical practice

Based on the findings of this COVID study, and future pandemics, nurse managers and supervisors should establish a culture of compassionate leadership to enhance positive nurse outcomes. Support groups for all nurses must be initiated to address their health and wellbeing, and work outcomes as to share their experiences and motivate others. Debriefing for all nurses should be compulsory with the focus on addressing work stressors and positive coping strategies, with open communication between managers and subordinates. Sessions could also be held to share information with nursing staff regarding services available and the right channels to follow when in need. Programmes such as Employee Assistance Programmes (EAPs)/Employee Wellness Programmes (EWPs) already assist, and awareness of these programmes should be promoted.

## Data Availability

The datasets analyzed during the current study are available from the corresponding author on reasonable request.

## Abbreviations

CFA: Confirmatory factor analysis
CPI: Compassion Practice Instrument
CSN: Community Service Nurses
COVID-19: Corona Virus Disease of 2019
EN: Enrolled Nurse
ENA: Enrolled Nurse Auxiliary
HSRC: Human Sciences Research Council
HREC: Health Research Ethics Committee
NRF: National Research Foundation
NWU: North-West University
NWU-HREC: North-West University Health Research Ethics Committee
PI: Principal investigator
PPE: Personal protective equipment
RN: Registered Nurse
RN4CAST: Registered Nurse Forecast
SA: South Africa
SPSS: Statistical Package for the Social Sciences
URL: Uniform resource locator
WHO: World Health Organization
